# Paid Caregiving in an Integrated System: Access to the Veterans Health Administration’s Home Health Aide Benefit

**DOI:** 10.1093/geroni/igaf122.1444

**Published:** 2025-12-31

**Authors:** Emily Franzosa, Lauren Hall, Margaret Ding

**Affiliations:** Bronx VA, Bronx, New York, United States; James J Peters VA Medical Center, New York, New York, United States; James J Peters VA Medical Center, New York, New York, United States

## Abstract

The Veterans Health Administration (VHA) is unique in offering home health aide services (HHA) at no or minimal cost to veterans. Serving 180,000 veterans, HHA is VHA’s fastest growing and most widely used home and community-based service. To understand factors affecting veterans’ HHA use, we conducted semi-structured, multi-perspective interviews with 60 key stakeholders at 5 geographically diverse VHA medical centers (10-15 per site, including VA HHA managers, primary care teams, and contracted home health agency administrators and aides). Interviews were analyzed through directed content analysis and translated to visual process maps to identify best practices, roadblocks and modifiable intervention points. We identified 4 key concepts: 1) HHA benefits were considered “part of a larger plan” including other VHA HCBS (respite, caregiver support, adult day, Veteran-directed care), Medicaid, or state programs, with VHA staff helping determine the most effective service mix; 2) some veterans paid privately for services VHA does not cover (e.g., transportation, housekeeping); 3) sites requiring veterans to verify income cited this as a barrier due to paperwork delays and veterans’ reluctance or inability to provide financial information; 4) some participants felt even minimal co-pays or fear of co-pays caused veterans to decline services, while others felt they increased veterans’ perceived value of the benefit. Our results suggest that navigating paid caregiving is complex even within an integrated health system, highlighting the need for close case management, patient education, reducing cost barriers, and closing gaps for those unable to pay for supplemental care.

